# Dr. George William Hackett

**Published:** 1915-10

**Authors:** 


**Dr. George William Hackett,** Buffalo 1895, was killed by his auto going over an embankment while turning out for a team, August 11. He was the father of Dr. Lawrence Hackett, a Buffalo graduate also.

